# Decoding Skin Aging Through Transcriptomic Clocks: Gene Expression Signatures, Associated Pathways, and Explainable AI

**DOI:** 10.3390/genes17050542

**Published:** 2026-05-01

**Authors:** Vasiliki Kefala, Vasiliki-Sofia Grech, Niki Tertipi, Eleni Sfyri, Apostolos Beloukas, Efstathios Rallis

**Affiliations:** Department of Biomedical Sciences, School of Health and Care Sciences, University of West Attica, GR-12243 Athens, Greece; valiakef@uniwa.gr (V.K.); ntertipi@uniwa.gr (N.T.); elsfiri@uniwa.gr (E.S.); abeloukas@uniwa.gr (A.B.); erallis@uniwa.gr (E.R.)

**Keywords:** transcriptomics, multi-omics, skin, aging, aging clocks, gene expression, fibroblasts, bulk RNAseq, single cell RNAseq, machine learning, explainable AI

## Abstract

Skin aging is a complex, multifactorial process driven by intrinsic biological mechanisms and environmental exposures, resulting in progressive functional and structural decline. Chronological age does not adequately capture this variability, highlighting the need for molecular biomarkers that reflect biological aging. In this context, transcriptomic aging clocks have emerged as a promising approach, as gene-expression profiles provide a dynamic representation of cellular and tissue states. This narrative review is based on a targeted literature search in PubMed and IEEE Xplore and focuses on transcriptomic aging clocks in human skin, with emphasis on gene-expression signatures, key biological pathways, and computational modeling strategies. These models consistently capture coordinated alterations in processes such as cellular senescence, DNA damage response, inflammation, and extracellular matrix remodeling. Representative transcriptomic frameworks, including models such as SkinAGE, illustrate the ability of gene-expression-based approaches to quantify biologically meaningful and dynamic aging states in the skin. Advances in machine-learning approaches, including deep learning and pathway-guided models, are critically evaluated, alongside the role of explainable artificial intelligence in enhancing model transparency and biological interpretability. Future developments are expected to integrate multi-omics data and digital twin frameworks, enabling the transition from static biomarkers toward dynamic, predictive, and personalized models of skin aging.

## 1. Introduction

Aging is a complex, multifactorial biological process characterized by progressive functional decline, increased susceptibility to disease, and reduced regenerative capacity. Although chronological age remains the most widely used measure of aging, it fails to capture the substantial interindividual variability observed in physiological decline and disease risk. Individuals of the same chronological age often exhibit markedly different molecular and functional states, reflecting the cumulative influence of genetic background, environmental exposures, and lifestyle factors. This discrepancy has driven the development of biological age estimators, which aim to quantify aging at the molecular level and provide a more accurate representation of an individual’s physiological state [1,2].

Among the various molecular approaches, omics-based aging clocks, including epigenomic, transcriptomic, proteomic, and metabolomic models, have emerged as powerful tools for quantifying biological aging. Currently, DNA methylation-based clocks demonstrate the highest accuracy in predicting chronological age; however, they primarily capture regulatory potential rather than active cellular function. In contrast, transcriptomic profiling reflects the actively expressed component of the genome, integrating upstream regulatory signals with downstream functional outcomes [3,4].

As such, transcriptomic data provide a dynamic and biologically interpretable layer of information that directly links gene regulation to cellular phenotype and tissue function. This concept is supported by multi-tissue transcriptomic studies demonstrating that conserved RNA expression signatures, including those observed in skin, can accurately capture biological aging and are associated with clinically relevant outcomes such as cognitive function and longevity [5].

Skin represents a particularly informative tissue for studying transcriptomic aging. As the largest organ and the primary interface with the external environment, it integrates intrinsic aging processes with environmental exposures, such as ultraviolet radiation, while also being shaped by endogenous processes, including oxidative stress and inflammation, which arise from both intrinsic cellular metabolism and external stimuli. These factors drive progressive remodeling of gene-expression networks, particularly in dermal fibroblasts, which orchestrate extracellular matrix homeostasis, tissue repair, and intercellular signaling. Consequently, skin exhibits robust, quantifiable, and biologically meaningful transcriptional changes that make it highly suitable for the development of tissue-specific aging models [6,7,8].

Moreover, advances in high-throughput sequencing technologies, combined with machine learning, have enabled the development of transcriptomic aging clocks, computational models that estimate biological age based on gene-expression profiles. By leveraging large-scale transcriptomic datasets, these models capture coordinated, system-level alterations in key biological pathways, including cellular senescence, DNA damage response, inflammation, and extracellular matrix remodeling, which collectively underpin the aging process. Importantly, while early transcriptomic clocks were primarily optimized for predictive accuracy, recent approaches increasingly incorporate explainable artificial intelligence (XAI) frameworks to enhance biological interpretability. Through these methods, it becomes possible to identify the specific genes and pathways that drive age predictions, thereby linking computational outputs to underlying molecular mechanisms. This shift from “black-box” prediction toward mechanistically informed modeling represents a critical step for the clinical applicability and biological translation of transcriptomic aging clocks [9,10,11,12].

Despite these advances, several key challenges remain. Transcriptomic clocks generally exhibit lower predictive accuracy compared to epigenetic models due to higher biological variability and sensitivity to environmental context. In addition, the extent to which these models reflect intrinsic aging versus disease-related or stress-induced transcriptional changes remains incompletely understood. Furthermore, the integration of transcriptomic data with other omics layers and the application of interpretable machine-learning frameworks are still evolving [4,9,11].

In this review, we aim to analytically examine transcriptomic aging clocks in human skin, focusing on the gene-expression signatures, biological pathways, and computational frameworks that underpin their development. We further explore the role of explainable artificial intelligence in enhancing biological interpretability and discuss how transcriptomic clocks contribute to understanding the molecular mechanisms of skin aging and disease-associated age acceleration. By integrating insights from transcriptomics, multi-omics, and artificial intelligence, this work provides a comprehensive framework for decoding biological aging in the skin.

## 2. Transcriptomics in Skin Aging

### 2.1. Transcriptome and Aging

The transcriptome encompasses the complete set of RNA transcripts produced from the genome within a cell or tissue at a defined physiological state, including messenger RNAs (mRNAs), ribosomal RNAs (rRNAs), transfer RNAs (tRNAs), long non-coding RNAs (lncRNAs), circular RNAs (circRNAs), and microRNAs (miRNAs). In contrast to the relatively static genome, the transcriptome represents the dynamic output of gene expression, providing a functional snapshot of cellular activity that reflects both intrinsic biological programs and environmental influences. As such, transcriptomic profiling enables direct interrogation of gene-regulatory networks that govern fundamental processes, including development, tissue homeostasis, and aging [13,14].

Gene expression, defined as the transcription of DNA into functional and regulatory RNA molecules, constitutes the primary mechanism through which genetic information is translated into cellular function. Because the transcriptome reflects the actively expressed component of the genome, transcriptomic analysis offers a uniquely dynamic and functionally relevant perspective on cellular physiology. In the context of aging, this is particularly important, as age-associated changes are not solely determined by genetic sequence but arise from cumulative regulatory, environmental, and metabolic influences that reshape transcriptional activity over time [13,14].

Accordingly, aging is characterized by progressive and systemic remodeling of the transcriptome, including increased transcriptional variability, shifts in RNA abundance, and reorganization of regulatory RNA networks. These alterations contribute to key hallmarks of aging, such as cellular senescence, impaired tissue regeneration, metabolic dysregulation, and chronic low-grade inflammation. Rather than occurring randomly, these transcriptional changes reflect coordinated activation and dysregulation of core biological pathways, including DNA damage response, cell-cycle control, inflammatory signaling, and extracellular matrix remodeling. Together, these processes establish a systems-level transcriptional landscape that underlies functional decline in aging tissues, particularly in the skin, where fibroblast-driven extracellular matrix remodeling plays a central role [13,14].

Beyond global gene-expression changes, transcriptomic studies have revealed complex RNA-mediated regulatory architectures that further shape aging-associated molecular signatures. In particular, competing endogenous RNA (ceRNA) networks, involving interactions between lncRNAs, miRNAs, and mRNAs, represent a critical layer of post-transcriptional regulation. For example, the lncRNA *RP11-670E13.6* modulates cellular senescence in UVB-exposed dermal fibroblasts by acting as a molecular sponge for miR-663a, thereby regulating the expression of cell-cycle genes such as *CDK4* and *CDK6* [15]. Additional regulatory axes, including *MEG3–miR-93-5p–epiregulin* and *H19–miR-296-5p–IGF2*, further illustrate how RNA networks coordinate inflammatory signaling, fibroblast function, and photoaging responses [16]. These findings highlight that transcriptomic regulation extends beyond linear gene expression changes to include multilayered regulatory interactions that directly influence aging phenotypes [15,16].

Importantly, transcriptomic alterations reflect the integration of multiple regulatory inputs, including transcription factor activity, chromatin organization, intercellular signaling, and environmental exposures such as ultraviolet radiation, alongside endogenous processes such as oxidative stress, which arise from both intrinsic metabolic activity and external stimuli. This integrative nature distinguishes transcriptomics from other molecular layers, as it represents the functional output of upstream regulatory mechanisms while remaining closely linked to downstream cellular phenotypes [4].

These properties position the transcriptome as a uniquely informative substrate for aging research, bridging molecular regulation and functional cellular state. Consequently, transcriptomic data provide a robust foundation for the development of computational models, including transcriptomic aging clocks, which aim to translate complex gene-expression patterns into quantitative measures of biological age [4].

Finally, transcriptomics should be considered within the broader multi-omics landscape of aging research. While epigenomics reflects regulatory modifications, proteomics captures functional protein activity, and metabolomics represents downstream biochemical states, transcriptomics occupies a central position by linking regulatory inputs to functional outputs. Integration of transcriptomic data with other omics modalities has further enabled the identification of robust aging-associated signatures, including genes such as *ETF1*, *PLBD2*, *ASAH1*, and *MOXD1*, highlighting the value of multi-layered approaches in improving biological interpretability [17].

### 2.2. Transcriptomic Technologies in Aging Research

Advances in high-throughput sequencing technologies have enabled the development of complementary transcriptomic approaches that are widely applied in aging research. Bulk RNA sequencing (RNA-seq) provides a comprehensive overview of gene expression across tissues or heterogeneous cell populations and has been extensively used to identify conserved transcriptional signatures and biological pathways associated with aging. Continuous methodological improvements, including short-read sequencing, long-read sequencing, and strand-specific RNA sequencing, have enhanced transcript detection and characterization, enabling more accurate quantification of both coding and non-coding RNA species and improving the resolution of transcript isoforms [13].

Despite its utility, bulk transcriptomics captures averaged gene-expression signals across mixed cell populations and therefore lacks the resolution to disentangle cellular heterogeneity. This limitation is particularly relevant in complex tissues such as the skin, where multiple cell types contribute to aging-related transcriptional changes. Single-cell transcriptomic technologies, including single-cell RNA sequencing (scRNA-seq) and single-nucleus RNA sequencing (snRNA-seq), overcome this constraint by enabling gene-expression profiling at the level of individual cells. These approaches allow the identification of cell-type-specific transcriptional programs and aging patterns that remain obscured in bulk analyses, thereby providing deeper insight into the cellular drivers of tissue aging [13].

In addition to single-cell approaches, spatial transcriptomics further expands transcriptomic resolution by integrating gene-expression data with spatial information within intact tissue architecture. This technology enables the mapping of transcriptional changes within their anatomical context, allowing the investigation of how aging-related molecular processes are distributed across tissue microenvironments and cellular niches. Together, bulk, single-cell, and spatial transcriptomic approaches provide a multi-scale framework for studying aging, ranging from global tissue-level signatures to cell-specific and spatially resolved regulatory dynamics. The main characteristics, resolution levels, and advantages of these approaches are summarized in Table 1 [13].

These complementary technologies form the methodological basis for dissecting both global and cell-type-specific transcriptomic changes in aging skin, as discussed in the following sections.

### 2.3. Global Signatures of Skin Aging

Large-scale transcriptomic studies have provided critical insight into how gene expression patterns change during human aging. In a comprehensive multi-tissue analysis, Glass et al. [18]. examined abdominal skin, subcutaneous adipose tissue, and lymphoblastoid cell lines, identifying 1672 genes in skin and 188 genes in adipose tissue whose expression levels were significantly associated with chronological age. These findings demonstrate that aging is accompanied by extensive transcriptional remodeling and highlight the skin as a tissue exhibiting particularly pronounced global transcriptomic signatures. Particularly, this study was based on bulk RNA sequencing, capturing averaged gene-expression profiles across heterogeneous cell populations and thereby providing a tissue-level overview of age-associated transcriptional changes [18].

Further functional enrichment analyses revealed that age-associated genes in skin are involved in key biological processes linked to tissue aging, including epidermal development, keratinization, epithelial differentiation, extracellular matrix organization, collagen fibril formation, fatty-acid metabolism, mitochondrial function, and RNA processing. Approximately half of these genes exhibited decreased expression with advancing age, whereas others were upregulated, reflecting process-specific transcriptional remodeling, whereby pathways involved in tissue structure and homeostasis decline, while stress- and inflammation-related pathways become increasingly activated. Notably, the overlap of age-associated genes across different tissues was limited, suggesting that transcriptional aging signatures are largely tissue-specific and reflect distinct regulatory programs operating within each tissue context [18].

Although these global transcriptomic signatures provide a foundational framework for understanding molecular aging at the tissue level, their interpretation is constrained by the inherent limitations of bulk profiling, which obscures cell-type-specific contributions. This limitation stresses the need for higher-resolution approaches, such as single-cell transcriptomics and integrative computational modeling, to dissect the cellular and molecular heterogeneity underlying skin aging.

### 2.4. Cell-Type Specific Transcriptomic Signatures Remodeling in Dermal Fibroblasts

Although tissue-level transcriptomic studies provide valuable insight into global aging signatures, skin aging is ultimately driven by molecular alterations occurring within specific cellular populations. Among these, dermal fibroblasts play a central role, as they are responsible for the synthesis and remodeling of extracellular matrix components that maintain dermal structure, elasticity, and mechanical integrity [19].

Transcriptome-wide analyses of primary human dermal fibroblasts derived from healthy individuals across a broad age range have revealed extensive age-associated remodeling of both coding and non-coding RNA landscapes. More than 1400 mRNAs and over 1000 long non-coding RNAs exhibit age-dependent changes in expression, reflecting widespread transcriptional reprogramming. Specific transcripts, including *OLFM1* and *FGF9*, show increased expression with advancing age, whereas others such as *RNFT2* and *WNT11* are downregulated. These coordinated alterations suggest progressive dysregulation of signaling pathways involved in cellular communication, extracellular matrix organization, and fibroblast functional capacity [19].

Transcription factor enrichment analyses further implicate members of the FOX and *HOX* families as potential upstream regulators of age-associated transcriptional programs in dermal fibroblasts, highlighting the role of higher-order regulatory networks in orchestrating transcriptional aging. Especially, the correlation between transcriptomic changes and protein abundance is modest, indicating that transcriptomic and proteomic data capture partially distinct and complementary layers of biological regulation. Despite this limitation, fibroblast-derived transcriptomic signatures provide critical molecular insight into dermal aging processes and represent a robust foundation for the development of biomarkers of biological skin age [19].

These findings emphasise the importance of cell-type-specific transcriptomic analysis, while also highlighting the need for higher-resolution approaches to capture cellular heterogeneity within aging skin.

### 2.5. Single-Cell Transcriptomics Signatures and Cellular Heterogeneity of Aging Skin

While transcriptomic analyses of isolated dermal fibroblasts provide important insights into cell-type specific aging processes, they still rely on bulk measurements that average gene expression across cellular populations and therefore cannot fully resolve cellular heterogeneity. In contrast, single-cell RNA sequencing (scRNA-seq) has enabled the characterization of transcriptional heterogeneity within aging skin at unprecedented resolution, allowing the identification of distinct cellular populations and state-specific transcriptional programs.

Application of scRNA-seq to human skin has substantially expanded understanding of the cellular complexity underlying skin aging. In a study by Zou et al. [20], single-cell transcriptomic profiling of eyelid skin from individuals across different age groups analyzed 35,678 individual cells and identified eleven distinct skin cell types, including keratinocytes, melanocytes, fibroblasts, endothelial cells, and immune cells, as well as six transcriptionally distinct subpopulations of epidermal basal cells. These findings highlight the extensive cellular diversity of human skin and provide a high-resolution framework for investigating age-associated molecular changes [20].

Combined morphological and transcriptomic analyses revealed that aging is associated with progressive structural and cellular alterations, including reduced epidermal thickness, decreased dermal collagen density, loss of specific cell populations such as endothelial cells, melanocytes, Langerhans cells, and pericytes, and accumulation of senescent cells in both epidermal and dermal compartments. At the molecular level, these changes are accompanied by cell-type specific transcriptional alterations, including age-related downregulation of developmental transcription factors such as *HES1* in dermal fibroblasts and *KLF6* in epidermal basal cells. Functional studies demonstrated that inactivation of these factors promotes cellular senescence and inflammatory responses, whereas activation of *HES1* alleviates fibroblast senescence, underscoring their regulatory role in maintaining skin homeostasis during aging [20].

Complementary single-cell analyses have further confirmed that aging is associated with a loss of fibroblast priming, characterized by reduced transcriptional specialization and diminished functional heterogeneity among fibroblast subpopulations. This shift toward a more homogeneous and less functionally distinct cellular state suggests that aging disrupts cell identity programs, thereby impairing the capacity of the dermis to maintain extracellular matrix homeostasis and respond effectively to environmental stress [9].

However, several limitations of single-cell transcriptomics should also be considered. Although this approach enables high-resolution characterization of cellular heterogeneity, it is often constrained by limited sample size and restricted anatomical sampling, which may reduce the generalizability of findings, particularly in a heterogeneous organ such as skin as in the present study. Here, the single-cell RNA sequencing was performed on eyelid skin samples from a relatively small cohort of donors across age groups, providing a controlled but anatomically and demographically restricted dataset [20]. In addition, the methodology requires enzymatic dissociation of tissue into individual cells, which disrupts native tissue architecture and results in the loss of spatial information, thereby limiting the ability to directly assess cell–cell interactions and microenvironmental organization [20].

Furthermore, single-cell RNA sequencing measures transcript abundance rather than protein expression or activity, and therefore does not capture post-translational modifications or functional cellular states. This limitation is particularly relevant in the context of skin aging, where key biological processes, such as extracellular matrix remodeling, inflammatory signaling, and DNA repair, are also regulated at multiple levels beyond transcription [20].

Finally, single-cell transcriptomic data represent static snapshots of gene expression, and aging-related processes are inferred through cross-sectional comparisons between age groups rather than direct longitudinal observation. Consistent with this, the study identifies progressive transcriptional changes, including increased inflammatory signaling and reduced proliferative capacity, but these conclusions are derived from comparative analyses rather than real-time tracking of cellular aging dynamics [20].

Overall, these limitations highlight that, despite its high resolution, single-cell transcriptomics should be interpreted within a broader integrative framework, ideally complemented by spatial transcriptomics, proteomics, and longitudinal approaches to fully elucidate the mechanisms underlying skin aging.

In this context, recent advances in skin spatial transcriptomics extend transcriptomic analysis beyond single-cell resolution by incorporating spatial tissue architecture and intercellular interactions. Although not specifically focused on aging, the primary goal of the study by Restrepo et al. [21]., was to comprehensively define the cellular and spatial organization of human skin at an organ-wide level. Using MERFISH-based spatial transcriptomics, the authors generated a large-scale spatial atlas encompassing approximately 1.2 million cells across 114 samples, 15 anatomical sites, enabling the identification of 45 distinct cell populations alongside their precise spatial localization. Crucially, this work demonstrated that human skin is organized into “microenvironments” which are not merely descriptive architectural features but constitute functionally active and dynamic units that undergo systematic remodeling in disease states. Specifically, integrative analysis across multiple inflammatory and neoplastic skin disorders revealed that distinct multicellular neighborhoods, most notably the PERIVASC I niche, are consistently expanded and transcriptionally reprogrammed, exhibiting enhanced immune-cell infiltration and signaling patterns reminiscent of tertiary lymphoid structures. Ligand-receptor analyses additionally highlighted key signaling pathways, such as TNF-mediated immune-stromal crosstalk through *CCL19^+^* perivascular fibroblasts [21].

Taken together, high-resolution transcriptomic analyses across tissues, cell types, and regulatory RNA networks provide a comprehensive molecular framework for understanding skin aging. These coordinated transcriptional alterations, encompassing cellular heterogeneity and tissue-specific regulatory dynamics, form the foundation for the development of transcriptomic aging clocks, which aim to quantitatively capture biological aging through gene-expression patterns.

## 3. Transcriptomic Aging Clocks

### 3.1. Concept and Development

Building upon these transcriptomic signatures of aging, transcriptomic aging clocks have emerged as computational models designed to estimate biological age from gene-expression data. These models are based on the premise that aging is accompanied by systematic and reproducible alterations in the transcriptome, including changes in RNA abundance, increased transcriptional variability, and modifications in transcriptional dynamics. Such coordinated transcriptional changes reflect cumulative shifts in cellular function and regulatory network activity, providing a biologically meaningful substrate for age prediction. By leveraging large-scale transcriptomic datasets and machine-learning approaches, transcriptomic aging clocks translate complex gene-expression patterns into quantitative measures of biological age, offering a functional and dynamic framework for assessing aging processes across tissues [13].

The first transcriptomic aging clock was introduced by Peters et al. in 2015 [22], who developed a predictive model based on gene-expression profiles from peripheral blood samples across large human cohorts. By analyzing transcriptomic data from thousands of individuals, the study identified 1497 genes whose expression levels were significantly associated with chronological age, implicating pathways related to metabolism, immune function, mitochondrial activity, and DNA repair. These gene-expression signatures were subsequently used to construct a predictor of “transcriptomic age,” enabling estimation of biological age from RNA profiles. Although the model demonstrated significant correlations with chronological age, its predictive accuracy was lower than that of DNA methylation–based clocks, partly due to technical variability arising from the integration of data generated across different platforms. Nevertheless, the transcriptomic predictor showed meaningful associations with aging-related phenotypes and risk factors, including smoking status and systolic blood pressure, suggesting that RNA-based clocks capture biologically relevant dimensions of aging that extend beyond chronological time [22].

Subsequent developments have further established transcriptomic aging clocks as a complementary approach to epigenetic age estimation. While DNA methylation–based clocks remain the most widely used biomarkers of biological age, transcriptome-based models provide a distinct perspective by capturing the actively expressed component of the genome. Changes in RNA abundance integrate multiple regulatory inputs, including transcription factor activity, chromatin organization, and environmental influences, thereby reflecting the functional state of cells and tissues during aging. Consequently, transcriptomic clocks offer a dynamic and mechanistically informative framework for quantifying aging processes and identifying gene-expression signatures associated with functional decline and disease susceptibility [23].

### 3.2. Skin as a Model Tissue for Transcriptomic Clocks

Skin represents a particularly informative tissue for transcriptomic aging-clock development, as its molecular and cellular characteristics are directly reflected in measurable gene-expression signatures associated with biological aging. As the largest organ and the primary interface with the external environment, the skin integrates intrinsic aging processes with environmental exposures, including ultraviolet radiation, while oxidative stress reflects both endogenous metabolic processes and responses to external stimuli, contributing to progressive remodeling of gene-expression networks [4,6].

At the cellular level, dermal fibroblasts play a central role in shaping these transcriptomic changes. As key regulators of extracellular matrix (ECM) homeostasis, fibroblasts control collagen synthesis, tissue remodeling, and intercellular signaling. With aging, fibroblasts undergo functional decline and cellular senescence, acquiring a senescence-associated secretory phenotype (SASP) characterized by pro-inflammatory cytokines, growth factors, and matrix-degrading enzymes. These processes are directly reflected in altered gene-expression profiles, making fibroblast-driven transcriptional remodeling a major contributor to age-associated transcriptomic signatures [7].

These alterations are mediated by key aging-related pathways, including *p53*, NF-κB, mTOR, and TGF-β signaling, which regulate cellular senescence, inflammation, and ECM dynamics. In parallel, oxidative stress, arising from both intrinsic cellular metabolism and environmental insults, activates transcription factors such as AP-1 and NF-κB, thereby promoting matrix metalloproteinase (MMP) expression and contributing to ECM degradation through a self-amplifying transcriptional feedback loop. Together, these coordinated pathway-level changes generate reproducible molecular signatures that can be captured by transcriptomic aging clocks [7,24].

At the tissue level, skin exhibits structured and multi-layered aging dynamics that are highly amenable to transcriptomic analysis. Notably, multi-omics studies integrating transcriptomic and epigenomic data have demonstrated that aging in human epidermis is not strictly linear but is characterized by distinct molecular stages associated with coordinated gene-expression changes and increased transcriptional variability. These stage-dependent alterations indicate that skin aging follows organized, biologically meaningful dynamics rather than random molecular drift, thereby enhancing the capacity of computational models to estimate biological age [25,26].

Particularly, the accessibility and regenerative nature of skin enable repeated sampling and longitudinal analysis, allowing transcriptomic approaches to capture dynamic changes in gene expression over time. Because transcriptomic profiles reflect ongoing cellular activity and regulatory network states, the skin provides a tissue in which age-related transcriptional remodeling is both pronounced and quantifiable. This makes it particularly suitable for developing transcriptomic aging clocks that not only estimate chronological age but also assess deviations in biological aging, such as the “age gap,” reflecting acceleration or deceleration of aging processes [25,27].

In this context, the age gap refers to the difference between predicted transcriptomic (biological) age and chronological age, providing a quantitative measure of whether tissue appears molecularly older or younger than expected. Positive values, indicative of age acceleration, have been associated with pathological processes including chronic inflammation and tissue damage, whereas negative values may reflect relative preservation of tissue function. Conversely, a negative age gap reflects decelerated aging, suggesting preserved tissue function or resilience [28,29].

To the best of our knowledge, the only potential example of transcriptomic age deceleration at skin has been reported in non-lesional skin of adalimumab-treated hidradenitis suppurativa patients, where gene-expression profiles appeared younger than expected based on chronological age in regression analyses. However, this observation was not consistent across analytical methods and no significant differences were detected in direct pre–post treatment comparisons [28]. Therefore, these findings should be interpreted with caution, as they likely reflect partial normalization of inflammation-driven transcriptional states rather than true reversal of biological aging.

Beyond skin, transcriptomic age has been shown to exhibit dynamic and reversible behavior in response to systemic perturbations. For example a recent work by An et al. [30], using whole-blood transcriptomic data demonstrated that transcriptomic age is highly dynamic and responsive to systemic perturbations. In this Korean cohort, acute COVID-19 infection was associated with marked transcriptomic age acceleration, reaching up to 21.31 years, during periods of homeostatic disruption, followed by a progressive return to baseline upon recovery. These observations reinforce the concept that transcriptomic age represents a functional and context-dependent biomarker, integrating the cumulative effects of intrinsic and extrinsic influences on molecular aging [30]. In this context, similar principles may be applicable to skin in the future, where transcriptomic profiling could provide insight into tissue-specific deviations from chronological aging.

Collectively, the combination of fibroblast-driven ECM remodeling, senescence-associated transcriptional reprogramming, environmentally responsive signaling pathways, and structured, stage-dependent tissue dynamics positions the skin as an optimal model system in which transcriptomic changes are sufficiently robust, interpretable, and biologically meaningful to support the development of accurate and tissue-specific aging clocks.

### 3.3. Transcriptomic Aging Models in Skin

#### 3.3.1. Early Fibroblast-Based Models

One of the earliest and most compelling demonstrations that transcriptomic data from human skin encode strong age-related information was provided by Fleischer et al. [31], whose primary objective was to determine whether gene-expression signatures in dermal fibroblasts could be used to predict chronological and potentially biological age. To this end, the authors generated a large RNA-seq dataset from dermal fibroblasts obtained from 133 healthy individuals spanning a broad age range (1–94 years), together with samples from patients with Hutchinson–Gilford progeria syndrome (HGPS), a model of accelerated aging [31].

Using an ensemble machine-learning framework based on staggered age-bin classifiers, this model achieved accurate prediction of chronological age, with a median absolute error of approximately 4 years, a mean absolute error of 7.7 years, and a coefficient of determination (r^2^) of 0.81, outperforming conventional regression-based approaches [31].

A central conceptual contribution of this study is the establishment of a baseline aging signal, derived from transcriptomic profiles of healthy individuals across the lifespan. By training the model exclusively on these samples, the authors define a normative reference of age-associated gene-expression patterns in dermal fibroblasts. This baseline enables the identification of deviations from typical aging, as demonstrated by the model’s ability to consistently predict higher ages in progeria samples compared to age-matched controls. These findings indicate that fibroblast transcriptomes capture biologically meaningful aspects of aging that extend beyond chronological time, supporting their utility as biomarkers of biological age [31].

Although the study does not explicitly define an “age gap,” the consistent observation that progeria samples are predicted as significantly older than age-matched controls reflects a systematic deviation between predicted and chronological age. This discrepancy can be interpreted as an early implicit representation of transcriptomic age acceleration, providing a conceptual foundation for the later formalization of age gap metrics in aging research [31].

Moreover, a key contribution of this work lies in establishing dermal fibroblasts as a suitable and informative substrate for transcriptomic aging models. Fibroblasts exhibit intrinsic properties that favor the preservation of aging-associated molecular signals, including low proliferative activity, accumulation of age-related damage, and well-characterized transcriptional and phenotypic alterations during aging. Importantly, these transcriptomic signatures are retained even after ex vivo culture, suggesting that the baseline aging signal is stable and can be reliably extracted through computational modeling [31].

However, based on the information provided in the study, no insights are offered regarding sex or ethnic dependent differences in transcriptomic aging. Although the dataset spans a wide age range, the analysis does not include stratification by sex, ethnicity, or other demographic variables, and therefore does not address population-specific variability in aging signatures [31].

Despite its strong predictive performance, this early model also presents several methodological limitations. The discretization of age into bins introduces systematic edge effects, leading to overestimation in younger individuals and underestimation in older age groups. Furthermore, while the model achieves high predictive accuracy, it provides limited biological interpretability, reflecting a broader limitation of early transcriptomic aging clocks, which prioritized predictive performance over mechanistic insight. Finally, although relatively large compared to earlier studies, the dataset size and age distribution may still influence model generalizability [31].

Generally, this study provides a proof of concept that transcriptomic aging clocks can be constructed from skin-derived cells and demonstrates that dermal fibroblasts represent a robust platform for capturing a stable, baseline aging signal. This work laid the foundation for subsequent models that aim not only to predict age but also to improve biological interpretability and clinical relevance [31].

#### 3.3.2. Deep Learning Models

Building on earlier fibroblast-based transcriptomic aging approaches, deep learning frameworks have been introduced to capture complex, non-linear relationships within high-dimensional gene-expression data. A representative example is the SkinAGE model developed by Xie et al. [10], which employs a deep neural network (DNN) to estimate aging-related transcriptomic patterns in human dermal fibroblasts. While the authors position the model as a tool for assessing biological aging and cellular senescence, it is important to note that the underlying architecture is trained in a regression framework to predict age-related outputs, which are subsequently interpreted as continuous “aging scores” rather than strictly chronological age [10].

The model was constructed through the integration of multiple dermal fibroblast RNA-seq datasets, followed by a multi-step feature selection pipeline combining differential expression analysis, weighted gene co-expression network analysis (WGCNA), and machine learning–based approaches, ultimately yielding a reduced set of 32 gene-expression features. Among nine machine learning algorithms evaluated, the DNN demonstrated the highest predictive performance (R^2^ ≈ 0.75, MAE ≈ 8.26, RMSE ≈ 10.91), indicating superior performance within the training framework. However, these performance metrics are derived from internally harmonized datasets, and external validation revealed a notable decline in accuracy (MAE ≈ 11.08 years), signifying limitations in cross-cohort generalizability [10].

A key conceptual contribution of the SkinAGE framework is the interpretation of model outputs as dynamic indicators of transcriptomic aging states. This is supported by experimental perturbation analyses, where UVB-induced photoaging increased predicted aging scores, while treatment with human embryonic stem cell-derived extracellular vesicles (hESC-EVs) reduced these values. Although these findings suggest that the model is sensitive to environmentally induced and intervention-related transcriptional changes, they should be interpreted cautiously, as such shifts may reflect modulation of stress-response and senescence-associated pathways rather than true reversal of biological aging [10].

At the molecular level, the observed transcriptomic signatures are enriched in pathways associated with cellular senescence and DNA damage responses, including *TP53* signaling and cell-cycle regulation. Key regulators such as *TP53*, *CDKN1A (p21),* and *CDKN2A (p16)* are implicated, consistent with established hallmarks of aging. Nevertheless, it remains unclear to what extent these features represent causal drivers of aging versus downstream markers of cellular stress, highlighting an inherent limitation in transcriptome-based inference [10].

The study also evaluates model performance across demographic subgroups, reporting lower prediction error in younger individuals and a tendency toward age underestimation in older samples, while no significant sex-based bias was observed. However, the absence of analyses across diverse ethnic or genetic backgrounds limits conclusions regarding broader population applicability [10].

From an interpretability perspective, the incorporation of SHAP (Shapley Additive exPlanations) enables post hoc identification of gene features contributing to model predictions. While this enhances transparency, it does not fundamentally resolve the “black-box” nature of the deep learning architecture, as interpretability is not intrinsically embedded in the model design [10].

Several additional limitations should be considered. First, the reliance on in vitro dermal fibroblast systems constrains the biological relevance of the model, as these do not recapitulate the cellular heterogeneity, spatial organization, or microenvironmental complexity of intact skin tissue. Second, transcriptomic profiles are inherently sensitive to transient environmental and experimental conditions, including culture state, UV exposure, and stress responses, which may confound aging-related signals. Third, although feature selection improves predictive efficiency, it may oversimplify the complex regulatory networks underlying aging, thereby limiting mechanistic insight [10].

Overall, while deep learning-based transcriptomic models such as SkinAGE represent a methodological advance in capturing high-dimensional gene-expression patterns, their outputs should be interpreted with caution. At present, these systems function primarily as sensitive detectors of transcriptional state changes rather than definitive measures of biological aging, and their clinical or translational applicability remains to be established.

#### 3.3.3. Interpretable Pathway-Guided Models

The limited interpretability of conventional transcriptomic aging models has prompted the development of approaches that explicitly embed biological knowledge within model architecture. A representative example is the knowledge-primed artificial neural network proposed by Holzscheck et al. [32], designed to couple age prediction with biologically interpretable representations of underlying molecular processes. Rather than relying on fully connected layers, the model constrains network architecture using predefined gene-pathway relationships, mapping gene-expression inputs from epidermal skin RNA-seq data to intermediate nodes corresponding to curated biological pathways, including the Hallmark gene sets [32].

This architecture enables prediction of chronological age with competitive accuracy (median absolute error ~4.7 years), albeit slightly lower than comparable fully connected “black-box” models trained on the same data, highlighting an inherent trade-off between interpretability and predictive performance. Importantly, the structured design allows intermediate neuron activations to be interpreted as proxy measures of pathway-level “aging states,” thereby providing a biologically grounded, albeit indirect, link between model output and functional processes [32].

At the systems level, pathway activation patterns demonstrate that transcriptomic aging is not driven by a limited set of dominant pathways but instead reflects widespread, coordinated alterations across diverse biological processes. Correlation-based ranking of pathway neuron activations with chronological age identified *TP53* and *TNFα/NF-κB* signaling among the most strongly associated pathways, with high correlation coefficients (~0.75–0.8)relative to a randomly constructed control pathway. Notably, differences in correlation strength across pathways were modest, supporting a distributed model of aging consistent with the concept of cumulative, multi-factorial molecular deterioration rather than single-pathway dominance [32].

The interpretability of this framework is further illustrated through in silico perturbation experiments, where simulated knockdowns of established aging-related genes (e.g., *SIRT1*, *TXNIP*) produced changes in predicted transcriptomic age consistent with prior biological knowledge. However, these findings primarily support the biological plausibility of learned associations rather than providing direct mechanistic or causal inference, as model outputs remain dependent on the statistical structure of the training data [32].

Despite these advances, several limitations remain. The reliance on predefined pathway annotations constrains discovery of novel regulatory mechanisms and introduces dependence on existing pathway definitions, which are often overlapping and context-specific. Furthermore, although interpretability is improved relative to conventional deep learning models, it remains partial, as pathway activations represent inferred rather than directly measured biological states. From a population perspective, the model is derived from a geographically defined cohort (SHIP-TREND), and its performance across diverse genetic and environmental backgrounds remains to be established [32].

Together, pathway-guided transcriptomic aging models represent an important conceptual advance by integrating predictive modeling with biologically structured interpretation. However, their outputs should be viewed as informative abstractions of pathway activity rather than definitive measures of biological aging, and further work is required to validate their generalizability and mechanistic relevance in vivo [32].

#### 3.3.4. Methodological Innovations

Beyond advances in model architecture and interpretability, recent developments in transcriptomic aging clocks have focused on improving predictive accuracy in the context of highly variable gene-expression data. A representative example is the BiT age framework developed by Meyer and Schumacher [23], which introduces a conceptually distinct methodological strategy for estimating biological age from transcriptomic data. The model is primarily constructed using *Caenorhabditis elegans* RNA-seq datasets encompassing diverse genetic backgrounds, environmental conditions, and lifespan-altering interventions, providing a controlled system for modeling biological aging dynamics, while human dermal fibroblasts are incorporated at a later stage exclusively to assess cross-species applicability [23].

The principal objective of the BiT age framework is to accurately predict biological age across heterogeneous conditions, rather than solely approximating chronological age. This is achieved through the integration of two key methodological innovations: temporal rescaling and transcriptome binarization. Temporal rescaling normalizes chronological age relative to the median lifespan of each experimental condition, enabling direct comparison between samples with markedly different lifespans and aligning them on a common biological scale. This approach allows the model to estimate biological age in absolute time units while accounting for lifespan variability across strains and treatments [23].

A central innovation of the framework is the binarization of gene-expression data. In this approach, continuous RNA-seq expression values are transformed into binary states based on whether the expression of each gene exceeds the median expression level within the same sample. This sample-specific thresholding reduces the impact of batch effects, sequencing depth variability, and technical noise, while retaining information about relative gene activity. As a result, the model becomes less dependent on absolute expression magnitudes, which are often inconsistent across datasets, and instead captures relative transcriptional patterns within each sample [23].

The combination of temporal scaling and binarization substantially improves predictive performance. The resulting model achieves near-theoretical accuracy in *C. elegans*, with an r^2^ of approximately 0.96, a Pearson correlation of 0.98, and a mean absolute error below one day, approaching the limits imposed by experimental variability in age annotation and lifespan measurements. Importantly, the model retains high accuracy across independent datasets and accurately reflects the effects of genetic mutations, environmental stressors, and pharmacological interventions, supporting its capacity to capture biologically relevant variation in aging [23].

In addition to its performance in model organisms, the underlying methodological framework was subsequently applied to human dermal fibroblast RNA-seq data, where binarization similarly improved age prediction accuracy (r^2^ ≈ 0.92). This extension serves as a proof-of-concept that the approach may be transferable to more complex biological systems, although it does not constitute a fully validated cross-species aging model [23].

Despite these advances, several limitations should be considered. The model relies on bulk RNA-seq data, which may obscure cell-type-specific aging signals and introduce biases related to population heterogeneity. Moreover, although binarization reduces technical variability, it simplifies continuous gene-expression information and may result in the loss of subtle quantitative differences. Finally, while temporal rescaling improves comparability across conditions, it assumes a uniform relationship between lifespan and aging rate, which may not fully capture non-linear or condition-specific aging dynamics [23].

Therefore, the BiT age framework represents a significant methodological advance in transcriptomic aging research by demonstrating that appropriate data transformation and normalization strategies can markedly enhance predictive performance [23].

#### 3.3.5. Clinical Applications

Recent work has begun to explore the application of transcriptomic aging clocks in clinically relevant contexts, particularly in inflammatory skin diseases. Jeremian et al. [28] investigated whether gene expression-based aging signatures derived from RNA-seq data of lesional and non-lesional human skin biopsies reflect disease-associated molecular alterations in conditions such as atopic dermatitis, hidradenitis suppurativa, and plaque psoriasis [28].

To address this, the authors applied seven transcriptional clock algorithms implemented within the RNAAgeCalc framework to publicly available datasets comprising patients and healthy controls. Of these, five algorithms (DESeq2, Pearson, Dev, GTExAge, and Peters) demonstrated moderate-to-strong correlations between predicted transcriptional age and chronological age across both healthy and diseased skin. Specifically, correlation coefficients ranged from r ≈ 0.40 to 0.86 in atopic dermatitis, 0.46 to 0.74 in hidradenitis suppurativa, 0.50 to 0.80 in psoriasis, and 0.32 to 0.60 in healthy controls (*p* < 0.05), indicating consistent but context-dependent predictive performance. However, predictive accuracy varied across conditions, with reduced performance observed particularly in lesional skin of hidradenitis suppurativa, likely reflecting increased transcriptional heterogeneity and disease complexity [28].

A central analytical feature of the study is the use of the age acceleration residual (AgeAccelResid), representing the deviation between predicted transcriptional age and chronological age. In contrast to methodological studies where such deviations are treated primarily as prediction error, here AgeAccelResid is interpreted as a potential indicator of disease-associated transcriptional shifts, although its biological and clinical significance remains to be fully established [28].

Using this framework, transcriptional age acceleration was generally observed in lesional skin compared with both healthy controls and matched non-lesional skin, although this effect was not uniform across all algorithms or disease contexts. The magnitude of age acceleration ranged approximately from +3.9 to +12.5 years depending on disease and analytical method, with stronger effects reported in psoriasis and hidradenitis suppurativa. In contrast, non-lesional skin showed more variable patterns, including instances of age deceleration, highlighting the context-dependent nature of transcriptomic aging signals [28].

Associations between transcriptional age acceleration and disease-related features were also identified. In atopic dermatitis, loss-of-function variants in the *FLG* gene were significantly associated with increased age acceleration, suggesting a link between barrier dysfunction and altered aging-related transcriptional profiles. Pathway enrichment analyses further demonstrated that clock-associated genes are enriched in biological processes related to inflammation, immune signaling, metabolism, and aging, including *TNF, IFNG*, *TGFB1*, and *NF-κB* signaling pathways. These findings support the notion that transcriptional age captures composite molecular signatures reflecting multiple interconnected biological processes [28].

From a clinical perspective, these results suggest that transcriptomic age acceleration may reflect aspects of disease-associated molecular remodeling and inflammatory burden. However, its interpretation as a biomarker requires caution. The study is based on cross-sectional data, limiting the ability to infer causality or temporal dynamics, and age acceleration was not consistently detected across all algorithms or disease states. Furthermore, the relationship between transcriptional aging signatures and disease progression, severity, or treatment response remains to be established [28].

Additional limitations include the use of heterogeneous public datasets, potential confounding by comorbidities and environmental factors, and limited population diversity, with most samples derived from individuals of European ancestry. The authors also emphasize that it remains unclear whether transcriptional age acceleration reflects intrinsic biological aging processes or adaptive transcriptional responses to disease states [28].

To sum up, these findings provide early evidence that transcriptomic aging clocks can capture disease-associated molecular alterations in human skin. While they highlight the potential of transcriptomic age as a research tool for linking inflammation and aging-related gene-expression patterns, further longitudinal, multi-cohort, and multiethnic studies will be required to establish their clinical utility and clarify the relationship between molecular aging and disease pathophysiology [28].

Taken together, the transcriptomic findings discussed in Section 2 and Section 3 converge on a set of recurrent gene-expression signatures and biological pathways that consistently underlie aging in human skin. While Section 2 highlights the mechanistic basis of transcriptomic remodeling, including fibroblast-driven extracellular matrix dynamics and regulatory RNA networks, Section 3 demonstrates how these molecular features are captured and quantified by transcriptomic aging models. Despite differences in computational architecture, these models consistently identify overlapping biological modules related to cellular senescence, DNA damage response, inflammation, barrier function, and extracellular matrix remodeling. This convergence indicates that transcriptomic aging clocks are grounded in biologically meaningful processes rather than arbitrary gene selection. To provide an integrated overview of these findings, Table 2 summarizes key skin-derived gene signatures, their associated pathways, and their mechanistic roles in aging.

It is clear by now that transcriptomic aging clocks capture multiple, biologically meaningful dimensions of aging, Nevertheless, while these models provide increasingly refined representations of biological aging, a fundamental limitation remains: the mechanisms through which individual gene-expression features contribute to model predictions are not inherently transparent. This challenge highlights the need for methodological approaches that bridge predictive performance with biological interpretability. In this context, XAI emerges as a critical framework for dissecting transcriptomic aging models, enabling the identification of key genes, pathways, and regulatory networks that shape age predictions and linking computational outputs to mechanistic insight. Last but not least, by enhancing model transparency and interpretability, XAI also supports trust, accountability, and clinical adoption in healthcare settings, where understanding the basis of algorithmic decisions is essential [33].

## 4. Explainable Artificial Intelligence in Transcriptomic Aging Models

The increasing use of complex machine-learning and deep learning architectures in transcriptomic aging clocks has significantly improved predictive performance but has simultaneously introduced a critical limitation: limited interpretability. Many high-performing models, particularly deep neural networks, operate as “black boxes,” making it difficult to determine which gene-expression features drive predictions and how these features relate to underlying biological mechanisms. This limitation is particularly important in biomedical applications, where transparency, reproducibility, and mechanistic insight are essential for clinical translation [11,33].

To address this challenge, XAI has emerged as a methodological framework that enables the interpretation of model predictions and facilitates the identification of biologically meaningful features. In transcriptomic aging clocks, XAI links model outputs, predicted biological age or aging scores, to input features, namely gene-expression levels, through feature-attribution methods. These approaches can be broadly classified into global and local explainability: global methods identify genes that consistently drive predictions across datasets, whereas local methods explain how specific gene-expression profiles influence the predicted age of an individual sample. Together, these strategies enable both system-level and patient-specific interpretation of transcriptomic aging signals [11].

In practice, most XAI applications in transcriptomic models rely on post hoc (extrinsic) explainability methods, meaning that explanations are generated after model training without modifying the underlying model. Methods such as SHAP and LIME (Local Interpretable Model-agnostic Explanations) estimate how variation in gene expression influences model output by assigning importance scores to individual features. For example, SHAP quantifies the marginal contribution of each gene to the predicted age by evaluating how predictions change when that gene is included or excluded across multiple feature combinations. This enables decomposition of transcriptomic aging predictions into contributions from specific genes and biological pathways, including cellular senescence, inflammation, and extracellular matrix remodeling. However, these explanations are derived from model behavior rather than internal structure and therefore represent approximations rather than direct mechanistic mappings [11,34,35,36].

This distinction highlights a fundamental conceptual separation between explaining a model and building an interpretable model. Post hoc XAI methods aim to interpret complex, pre-trained models, whereas intrinsically interpretable models incorporate biological structure directly into their architecture, enabling predictions to be understood without external explanation techniques [11,34].

Within transcriptomic aging research, the SkinAGE model represents a clear example of post hoc XAI application. A deep neural network is used to predict aging-related transcriptional states, and SHAP is subsequently applied to attribute these predictions to individual genes and pathways, including regulators of *TP53* signaling and cell-cycle control. While this approach provides biologically relevant insights, the model itself remains a black box, and the explanations reflect estimated feature contributions rather than the true internal decision process. Thus, SkinAGE incorporates XAI but does not achieve full transparency or mechanistic interpretability [10,11].

Alongside, the pathway-guided neural network developed by Holzscheck et al. [32], represents an intrinsically interpretable approach. By embedding predefined gene-pathway relationships into the model architecture, predictions can be directly expressed in terms of pathway activity, allowing a more immediate biological interpretation of transcriptomic aging. However, this approach does not rely on XAI methods and therefore is not considered XAI in the strict sense. Moreover, its interpretability is constrained by prior biological knowledge and may not capture novel or uncharacterized regulatory interactions [32].

Taken together, these approaches illustrate that current transcriptomic aging models remain limited in their ability to fully explain how gene-expression patterns determine biological age. Post hoc XAI provides indirect, approximate explanations of black-box models, whereas intrinsically interpretable models improve transparency but at the cost of flexibility and discovery. Consequently, existing frameworks represent intermediate steps along a continuum of interpretability rather than fully explainable systems [11].

Advancing toward truly interpretable transcriptomic aging clocks will require the integration of predictive performance with models that more directly reflect underlying biological mechanisms, thereby enabling a more faithful mapping between gene-expression features, regulatory pathways, and aging phenotypes [11,12].

## 5. Future Directions: Limitations, Clinical Translation, and the Transition to Integrative Transcriptomic Aging Models

Despite substantial methodological advances, the overall accuracy and practical utility of transcriptomic aging clocks remain context-dependent and should be interpreted with caution. Early fibroblast-based models, together with more recent machine learning approaches, demonstrate moderate-to-strong predictive performance (e.g., r^2^ values ranging from ~0.75 to 0.81) [10,31,32], with some frameworks achieving higher correlations (e.g., r^2^ ≈ 0.92 in binarization-based models applied to human fibroblasts) [23]. However, these improvements are often model- and dataset-specific and do not consistently translate into reduced prediction error across independent cohorts.

Principally, correlation-based metrics such as r^2^ primarily capture the strength of linear association with chronological age and may overestimate model performance, whereas MAE provides a more clinically relevant measure of predictive accuracy. In this background, transcriptomic clocks generally exhibit higher MAE values (~4–8 years) compared to DNA methylation–based clocks (~3–5 years), which remain the most accurate molecular estimators of chronological age [37]. This discrepancy reflects fundamental biological differences between the two approaches: DNA methylation profiles represent relatively stable regulatory marks, whereas transcriptomic data capture a dynamic and environmentally responsive layer of gene expression [4]. Accordingly, variability in transcriptomic predictions should not be viewed solely as a limitation but also as an intrinsic property, as these models reflect transient cellular states influenced by inflammation, stress, and experimental conditions.

Furthermore, variability across datasets, tissues, and disease contexts, together with limited generalizability and the absence of standardized analytical frameworks, stresses that these models remain largely exploratory. This is exemplified by studies in inflammatory skin diseases, which demonstrate heterogeneous performance and context-dependent age predictions across algorithms and clinical states [28]. Therefore, although transcriptomic clocks provide biologically meaningful insights into aging-related molecular processes, their clinical applicability and reliability as standalone biomarkers are not yet fully established. Mutually, these observations highlight a critical gap between model performance in controlled experimental settings and their behavior in real-world clinical environments, where biological complexity and heterogeneity substantially challenge predictive accuracy.

Building on these considerations, the next generation of transcriptomic aging research is expected to move beyond single-layer, predominantly descriptive models toward integrative, mechanistically informed, and clinically actionable frameworks. This transition will likely be driven by the convergence of multi-omics integration, high-resolution transcriptomic technologies, explainable artificial intelligence (XAI), and emerging digital twin paradigms.

### 5.1. Spatial Resolution of Aging Processes

The integration of spatial and single-cell transcriptomic technologies into aging-clock frameworks represents a key step toward improving biological resolution and contextual accuracy. Unlike bulk RNA-seq, which averages gene expression across heterogeneous cell populations, these approaches enable the identification of cell-type-specific transcriptional changes and preserve spatial information within intact tissue architecture. This added resolution is particularly important for skin, where aging and disease processes are shaped by complex interactions between distinct cell populations and their local microenvironments [38].

Spatial transcriptomics, in particular, provides insight into how gene-expression patterns are organized within tissue and how cellular neighborhoods contribute to aging-associated molecular changes. A representative example is the study by Restrepo et al. [21], which generated a high-resolution spatial atlas of human skin and demonstrated that tissue “microenvironments” are dynamic and functionally active units. Their findings show that spatially organized cellular interactions and signaling networks play a central role in tissue remodeling, linking molecular alterations to tissue-level changes. This framework enables more precise characterization of disease-associated processes and supports the identification of spatially restricted molecular targets, thereby offering a more context-aware understanding of skin aging and pathology in the future [21].

### 5.2. Clinical Translation

The clinical relevance of transcriptomic approaches is increasingly evident, particularly in dermatology, where skin-derived molecular data can be directly linked to disease mechanisms and therapeutic outcomes.

In atopic dermatitis, a large-scale longitudinal study combining RNA sequencing from minimally invasive 1-mm skin biopsies with circulating cytokine profiling identified distinct gene-expression signatures corresponding to specific inflammatory endotypes and skin phenotypes, which closely correlate with disease severity. This approach further revealed associations between baseline transcriptomic profiles and therapeutic response to dupilumab, with persistent type 17-skewed transcriptional programs observed in poor responders. These findings accentuate the value of integrating tissue-specific transcriptomic data with systemic biomarkers to refine disease phenotyping, support patient stratification, and enable long term assessment of treatment response within a precision dermatology framework [39].

In oncology, transcriptomic profiling has emerged as a powerful tool for dissecting the molecular architecture of skin cancers, enabling the characterization of tumor heterogeneity, microenvironmental interactions, and clinically actionable biomarkers. Integrated transcriptomic analyses have identified key features of tumor biology, including immunosuppressive microenvironment, such as reduced T-cell infiltration in melanoma, as well as distinct malignant cell subpopulations and signaling pathways that drive tumor progression, immune evasion, and therapeutic resistance. In parallel, these approaches have facilitated the identification of clinically relevant biomarkers and therapeutic targets, including MITF/AXL transcriptional states in melanoma and cancer-associated fibroblast (CAF) subtypes in cutaneous squamous cell carcinoma, thereby supporting precision diagnosis, patient stratification, and the development of targeted therapies [40]. Mutually, these advances highlight the translational potential of skin transcriptomics as a bridge between molecular characterization and clinical decision-making.

Beyond oncology, studies in other tissues further emphasize the importance of microenvironmental context in aging and disease. Spatially resolved single-cell transcriptomic analyses in brain tissue have demonstrated that neighboring cells can significantly influence each other’s aging state through cell–cell proximity effects, revealing a previously unrecognized layer of tissue-level aging regulation [41].

Nevertheless, transcriptomic aging clocks extend these applications by providing quantitative measures of biological aging. In skin, Jeremian et al. [28], demonstrated that lesional tissue from patients with inflammatory dermatoses exhibits significant transcriptional age acceleration compared to both non-lesional skin and healthy controls. Similarly, in human blood, accelerated transcriptomic aging has been associated with upregulation of inflammatory and immune pathways, downregulation of metabolic processes, and increased morbidity risk, particularly in chronic conditions such as HIV infection [42]. Whereas, pronounced age acceleration during acute disease states, such as COVID-19, is followed by a return toward baseline upon recovery, reflecting restoration of transcriptional homeostasis [30].

Taken together, these findings position aging transcriptomics as a functional biomarker of inflammatory burden and tissue-level aging dysregulation, with potential applications in disease stratification, monitoring of therapeutic response, and the advancement of precision dermatology.

### 5.3. Multi-Omics Integration

Future developments in transcriptomic aging clocks are expected to advance toward integrative multi-omics frameworks that more accurately capture the complexity of biological aging. By combining complementary molecular layers, such approaches provide a more comprehensive representation of aging processes than single-omic models.

An illustrative example is the study by Holzscheck et al. [25], which integrated transcriptomic and epigenetic data in human epidermis to investigate the structure and dynamics of aging. Using a longitudinal design with repeated sampling of the same individuals, the study demonstrated that aging follows non-linear, stage-dependent transitions that correlate more closely with biological than chronological age. These transitions were characterized by progressive transcriptional dysregulation, increased molecular heterogeneity, and reduced coordination of pathway activity, highlighting the importance of multi-omics approaches for capturing dynamic, tissue-specific aging patterns [25].

Complementary evidence comes from large-scale multi-omics analyses in human blood, where integration of epigenetic and transcriptomic data across multiple cohorts enabled the identification of “multi-omic aging genes” enriched in adaptive immune pathways. These signatures showed improved reproducibility across populations and stronger associations with aging-related outcomes, including mortality risk, compared to single-omic models. In addition, they highlighted regulatory loci with potential relevance for epigenetic modulation, suggesting possible targets for interventions aimed at modifying aging-related processes [43].

These studies highlight the paired strengths of multi-omics approaches: longitudinal and tissue-specific resolution in skin, and cross-population robustness in blood. The integration of such approaches through advanced computational frameworks is likely to enhance both predictive performance and biological interpretability of aging models across tissues [25,43].

### 5.4. Hybrid Artificial Intelligence Approaches and Digital Twins Towards Predictive Precision Dermatology

The realization of these integrative frameworks will depend critically on advances in explainable and mechanistically informed artificial intelligence. Current transcriptomic aging models often rely on either high-performance “black-box” architectures or biologically constrained but less flexible interpretable models. Future approaches are likely to adopt hybrid strategies that combine data-driven learning with structured biological knowledge, such as pathway-guided or network-based architectures. These models would enable simultaneous optimization of predictive performance and biological interpretability, facilitating the identification of key regulatory pathways and potential therapeutic targets [11,32].

Moreover, digital twins as computational representations of individual biological systems could incorporate transcriptomic, epigenomic, proteomic, and clinical data to reconstruct personalized aging patterns and predict responses to environmental exposures, such as ultraviolet radiation, or therapeutic interventions. Notably, the integration of genetic, real-time biological, environmental, and lifestyle data, combined with artificial intelligence and digital phenotyping, enables continuous model updating and enhances the capacity to simulate disease progression and treatment outcomes in a patient-specific manner. This approach would extend transcriptomic aging clocks from static estimators of biological age to dynamic, predictive systems capable of modeling temporal changes in tissue state [12,44,45].

### 5.5. Expanding the Scope

Emerging transcriptomic aging clocks enable biological age prediction at the resolution of individual cell types across multiple human tissues. Leveraging large-scale datasets, these models, demonstrate improved predictive performance, with nearly half of tissue-level cell types achieving mean absolute errors below 10 years, while also revealing substantial variability in aging patterns across cell types. Notably, skin-derived cell types exhibit among the highest levels of transcriptomic age acceleration, as reflected by elevated Aging Deviation Index (ADI) values, indicating pronounced aging-related transcriptional changes compared to other tissues. Although these findings require further validation, they highlight the sensitivity of skin as a model tissue for detecting biologically relevant aging processes and support the use of cell-type resolved approaches for linking molecular aging signatures to disease-associated alterations and functional cellular states [46].

Building on this tissue sensitivity, the skin may also serve as a window into systemic aging processes. Given the shared ectodermal origin of the skin and central nervous system, together with the documented expression of neurodegeneration-associated genes and proteins in cutaneous tissues, transcriptomic alterations in the skin may reflect broader, system-level or CNS-related pathological changes [47]. Conceptually, this raises the possibility that skin-based transcriptomic aging models could be developed as minimally invasive tools for capturing early molecular signatures associated with neurodegenerative diseases. While this proposition remains speculative at present and is not yet supported by direct evidence, it is biologically plausible and may become testable with ongoing advances in high-resolution transcriptomics, multi-omics integration, and artificial intelligence-driven modeling frameworks.

## 6. Conclusions

Transcriptomics and transcriptomic aging clocks establish a grounded framework for quantifying skin into measurable indicators of biological age. By capturing coordinated alterations across key pathways, including cellular senescence, DNA damage response, inflammation, and extracellular matrix remodeling, these models provide a pathway-level molecular readout of tissue state, directly linking transcriptional activity to functional decline.

Importantly, transcriptomic age reflects a context-dependent and integrative signal shaped by intrinsic aging processes, environmental exposures, and disease-associated remodeling. Within this framework, age acceleration emerges as a clinically relevant indicator of tissue dysfunction and inflammatory burden, although its interpretation remains constrained by biological variability, limited standardization, and incomplete mechanistic transparency.

Future progress will depend on the integration of transcriptomic clocks with multi-omics data, spatial and single-cell technologies, and explainable artificial intelligence. These advances are expected to transform transcriptomic aging models from descriptive tools into predictive and clinically actionable systems, enabling longitudinal monitoring of tissue aging, early detection of pathological deviations, and the development of personalized therapeutic strategies.

## Figures and Tables

**Table 1:** Three main transcriptomic approaches used in aging research [13].

Transcriptomic Type	Resolution	Main Advantage in Aging Research
Bulk RNA-seq	Tissue level	Identifies global aging signatures and pathways
Single-cell RNA-seq/snRNA-seq	Individual cell level	Reveals cellular heterogeneity and cell-type-specific aging
Spatial transcriptomics	Cells within tissue context	Maps where aging processes occur within tissues

**Table 2:** Skin-Specific Gene Expression Signatures and Mechanistic Pathways in Transcriptomic Aging Model.

Biological Module	Skin-Derived Gene Signature	Associated Pathways	Mechanistic Role in Skin Aging	Source
Cellular senescence, cell-cycle arrest (fibroblasts)	*CDKN1A*, *CDKN2A*, *TP53*	TP53 signaling	Induction of fibroblast senescence, growth arrest, SASP activation contributing to dermal aging	[7,10]
DNA damage response (photoaging & intrinsic aging)	*TP53*, *PARP1*	DNA damage response pathways	Response to UV-induced and oxidative DNA damage; activation of repair or senescence pathways in skin cells	[10,24]
Inflammation, immune activation (skin disease & aging)	*TNF*, *IFNG*, *TGFB1*	TNFα/NF-κB signaling; TGF-β signaling	Chronic inflammation (inflammaging), immune activation, fibrosis, and dermal remodeling	[28,32]
Barrier dysfunction (epidermis-specific)	*FLG*	Epidermal differentiation/barrier pathways	Impaired skin barrier: increased inflammation and accelerated transcriptional aging	[28]
Extracellular matrix remodeling (dermis)	(MMP regulation via AP-1/NF-κB)	TGF-β, AP-1, NF-κB pathways	Collagen degradation, reduced ECM synthesis, loss of dermal integrity and elasticity	[7,24]
Fibroblast-specific transcriptional remodeling	*OLFM1*, *FGF9*, *RNFT2*, *WNT11*	Developmental signaling/ECM-related pathways	Altered fibroblast communication, ECM organization, and regenerative capacity with age	[19]
Transcriptional regulation in skin cells	FOX family, *HOX* family transcription factors	Gene regulatory networks	Upstream control of age-associated transcriptional programs in dermal fibroblasts	[19]
Integrated skin aging biomarkers (multi-omics validated)	*ETF1*, *PLBD2*, *ASAH1*, *MOXD1*	Cross-omics regulatory networks	Robust skin aging markers validated across transcriptomic and proteomic layers	[17]

## Data Availability

No new data were created or analyzed in this study. Data sharing is not applicable to this article.

## Abbreviations

Shapley Additive Explanations (SHAP), Ultraviolet (UV), Explainable Artificial Intelligence (XAI), Senescence-Associated Secretory Phenotype (SASP), LIME (Local Interpretable Model-Agnostic Explanations), Extracellular Matrix (ECM), Matrix Metalloproteinase (MMP), Mean Absolute Error (MAE).
